# Pernicious Anæmia

**Published:** 1905-08-12

**Authors:** 


### Hospital Clinics.

### PERNICIOUS ANÆMIA.

The June issue of the "Johns Hopkins Hospital Bulletin" contains an interesting communication from Dr. C. H. Bunting on the ætiology and pathogenesis of pernicious anæmia. This author states that there is probably no particular racial liability to the disease, nor any noticeable preponderance of either sex among its victims. The age-period supplying the bulk of instances is that of middle life, and the patients are generally people of previously robust habit, and inclined to be obese.

The question whether the symptoms and blood-picture which are commonly accepted as indicating pernicious anæmia actually represent a distinct disease is still *sub judice*, as it has been any time these 30 years. Addison, who first described these symptoms in 1855, thought the disease a distinct one. He speaks of "a very remarkable form of general anæmia occurring without any discoverable cause whatever. . . . The disease presented in every instance the same general character, pursued a similar course, and with scarcely a single exception, was followed after a variable period by the same fatal result." Biermer, in 1872, reported 15 cases of anæmia with the general symptoms of pernicious anæmia, and reached the conclusion that the phenomena mentioned were dependent upon a variety of causes. The same line of cleavage marks professional opinion to-day, German authorities following the tenets of Biermer, while English and American physicians follow the teaching of Addison. Thus William Hunter, a prominent disciple of the English school, strongly upholds the specific nature of the disease in the following words:—"The anæmia which Addison described is a remarkable infectious disease with definite antecedents, mode of origin, infective and hæmolytic lesions, and course." He adds that the studies which led him to this conclusion left it clear to his mind that Biermer's cases illustrated an entirely different nosological group which owed their origin to septic conditions, especially of the alimentary canal.

Dr. Bunting, after a comprehensive review of the various suggestions from time to time put forward in explanation of the occurrence of pernicious anæmia, records some important researches by William Hunter directed to prove that the fundamental feature of pernicious anæmia is a hæmatolytic process in the sphere of the portal circulation. This observer made comparative observations on the quantity of iron present in the liver and spleen respectively after death from pernicious anæmia in the one case, and secondary anæmias in the other. He found that in seven subjects dying of pernicious anæmia the liver and kidneys contained 360 milligrammes of iron per 100 grammes of the dried organs, while the spleen contained only 125 milligrammes of iron to the same weight of substance. In seven secondary anæmias, on the other hand, the figure for the spleen was 362 milligrammes, as opposed to only 79 for the liver and kidneys. This striking variation in the deposits of iron in the two conditions points strongly, in Hunter's opinion, to an excessive erythro-cytolysis in the liver, or at all events in the portal circulation, and suggests the action of a hæmolysin derived from abnormal intestinal processes. In this connection it is noteworthy that hæmolysins have been identified with certain intestinal parasites, particularly the bothriocephalus worm.

Dr. Bunting inclines to think that this absorption of some intestinal toxin is the basis of the disease, but that it acts by determining a faulty hyperplasia of the marrow. The case for a toxic process is assisted by the occasional occurrence of symmetrical lesions in the nervous system, comparable to those appearing in such intoxications as ergotism, pellagra, and lathyrism. Dealing with the nature of the toxin and its mode of action, the author expresses his conviction that Ehrlich is in error in asserting that the megaloblast is not a normal constituent of marrow, for the former has found it in the marrow of all the common laboratory animals and in man. He states that there is in the marrow a definite arrangement of both leucocytic and erythrocytic elements. He has identified nests or groups of cells to which he gives the name of erythrogenetic and leucogenetic centres. In the case of the former, the centre of the group is occupied by megaloblasts; traced towards the periphery of the group the type of cell becomes that of a nucleated red of smaller size, but still with a vesicular nucleus, then that of a normoblast with a pyknotic nucleus, and finally, at the extreme periphery, that of a mature non-nucleated red cell. Similarly in the leucogenetic centres, the central zone is occupied by myelocytes, the periphery by polymorphonuclear leucocytes. The normal development of the blood appears therefore to proceed as follows: A division of the central megaloblast produces either a similar cell or a smaller one, "the intermediate" type of nucleated red-cell. By reproduction of these the normoblast makes its appearance, and lastly the mature non-nucleated cell. As a result of this grouping the more mature cells are constantly being extruded towards the periphery of the group, and thus the maturest cells are in closest apposition to the capillaries, and are likely to be included in the first draught upon the hæmopoietic system in an emergency.

The reaction of this system to stimuli varies within wide limits according to the nature of the stimulus. In the simplest event, namely the depletion attending a gross hæmorrhage, the tendency of the response is well indicated by the following experiments carried out by Dr. Bunting. A rabbit was bled from one of the veins of the ear almost daily for 18 days. The average daily withdrawal of blood amounted to 10 cubic cm., and the bleeding resulted in a reduction of the red-cells from 5,988,000 per cubic mm. to 2,486,000. Twenty-four hours after the bleeding, examination never showed more than 16 normoblasts per cubic mm. though after a shorter interval (two hours), the figure rose as high as 108. But the result of injection of myelotoxic sera was very widely different. The serum of a rabbit immunised against the red blood-corpuscles of a dog, when injected into a dog brought into the circulation 24 hours later no fewer than 900 normoblasts and 120 megaloblasts per cubic mm. After 48 hours the normoblasts remained in approximately the same excess, but the megaloblasts fell to 64. After 72 hours the normoblasts reached 500 while megaloblasts had disappeared. For subsequent experiments ricin was used in place of the myelo-toxic serum. This substance has a marked hæmolytic effect upon the blood of rabbits, and gave the following results:—Twenty-four hours after the injection of a lethal dose of ricin, the blood of the injected animal gave a count of 1,130 normoblasts per cubic mm., 603 naked pyknotic red-cell nuclei, and 335 megaloblasts. Administration of a smaller dose gave rise, in the first instance, to the appearance of normoblasts without megaloblasts, but a repetition of the dose brought megaloblasts into the circulation. The author observes that after a small dose of ricin the blood-picture bears a remarkable resemblance to that of pernicious anæmia.

Dr. Bunting concludes that in the simple case of red-cell deficiency depending upon hæmorrhage, the draught upon the hæmopoietic system is met in the first instance by the reserve of mature red-cells, supplemented, if the deficiency is extreme, by a few relatively immature normoblasts. But when the matter is one of hæmolysis by a circulating poison, which not only destroys the current erythrocytes, but acts injuriously upon the manufactory itself in the marrow, the emergency is greater and the call for material so insistent that the immaturest cells are shed into the circulation. The moral of this theory is that in any given case of pernicious anæmia the gravity of the prognosis varies directly with the numerical bulk of megaloblasts called upon for immediate service in the circulation.

